# Validation of Falls Efficacy Scale Instrument in Romanian Adults with Type 2 Diabetes Mellitus: A Monocenter, Prospective Study

**DOI:** 10.3390/diagnostics16081135

**Published:** 2026-04-10

**Authors:** Bianca Iliescu, Andreea Herascu, Laura Gaita, Vlad-Florian Avram, Adina Braha, Bogdan Timar

**Affiliations:** 1 Doctoral School of Medicine, “Victor Babes” University of Medicine and Pharmacy, 300041 Timisoara, Romania; bianca.iliescu@umft.ro; 2 Sebes Municipal Hospital, 515800 Sebes, Romania; 3 Centre for Molecular Research in Nephrology and Vascular Disease, “Victor Babes” University of Medicine and Pharmacy, 300041 Timisoara, Romania; gaita.laura@umft.ro (L.G.); avram.vlad@umft.ro (V.-F.A.); braha.adina@umft.ro (A.B.); bogdan.timar@umft.ro (B.T.); 4 Department of Diabetes, “Pius Brinzeu” Emergency Hospital, 300736 Timisoara, Romania; 5 Second Department of Internal Medicine, “Victor Babes” University of Medicine and Pharmacy, 300041 Timisoara, Romania

**Keywords:** type 2 diabetes, diabetic neuropathy, fear of falling, FES-I

## Abstract

**Background**: Fear of falling is common in older adults with type 2 diabetes mellitus (T2DM), particularly in those with balance and mobility impairment. The Falls Efficacy Scale—International (FES-I) is widely used to quantify concern about falling but requires local validation. We aimed to validate the Romanian version of the FES-I in older adults with T2DM. **Methods**: In this validation study, 124 consecutive outpatients with T2DM aged > 60 years completed the Romanian FES-I at baseline (v1) and at one-month follow-up (v2). Internal consistency was assessed with Cronbach’s alpha and item–total correlations. Test–retest reliability was evaluated using intraclass correlation coefficient (ICC) and the Bland–Altman agreement. Construct validity was examined by correlations with the Fear-of-Falling Questionnaire—Revised (FFQ-R), Berg Balance Scale (BBS), Timed Up and Go (TUG), and single-leg stance (SLS). Discriminative performance was assessed via ROC analyses. **Results**: Mean FES-I scores were 30.8 ± 11.4 (v1) and 31.1 ± 11.6 (v2). Internal consistency (Cronbach’s alpha 0.945–0.947) and test–retest reliability (ICC 0.972; 95% CI 0.956–0.983) were excellent, with minimal bias. FES-I correlated strongly with FFQ-R (rho = 0.787) and moderately with function (BBS rho = −0.631; TUG rho = 0.547; SLS rho = −0.498; all *p* < 0.001). Discrimination was good for BBS (AUROC = 0.779) and TUG (AUROC = 0.800). **Conclusions**: The Romanian FES-I demonstrates excellent reliability and good validity in older adults with T2DM, with low measurement error and clinically interpretable change thresholds. It can be used for fear-of-falling quantification in routine care and research, including longitudinal monitoring and evaluation of interventions in Romanian patients with diabetes.

## 1. Introduction

Diabetic neuropathy (DN) is a common complication of type 2 diabetes mellitus (T2DM), affecting approximately one in two patients [1]. It may cause impaired balance and gait, neuropathic pain, and reduced functional capacity, while often remaining difficult to manage with conventional treatment strategies [2]. These features highlight the need for early screening and implementation of targeted interventions.

It is important to evaluate the combination of DN signs, symptoms, and severity, as well as the patient’s perception of balance problems, even among individuals who are not yet considered at high risk of falls. In people with diabetes, these factors may be more relevant than age-related physical impairments alone [3], because DN is associated with both motor nerve dysfunction and peripheral sensory damage [4].

Controlling foot trajectory to achieve safe clearance from the ground is one of the key motor tasks during gait [5]. Reduced foot clearance during the swing phase is directly associated with a higher risk of tripping and falling [6].

Falls and the fear of falling are frequent in patients with T2DM, especially in elderly patients. Both are influenced by neuropathy, autonomic disorders and other comorbidities (coronary artery disease, retinopathy, obesity and arthritis) [7]. DN severely affects quality of life causing bone fracture, inadequate healing wounds, amputation and mobility impairment [8].

Falls among older adults are common, may have serious consequences for the affected person, and are associated with substantial costs for healthcare systems [9].

Fear of falling is defined as “a persistent worry about falling that causes a person to avoid activities that he or she remains capable of performing” [10]. Fear of falling may exist even in the absence of previous falls and can lead to activity avoidance, inability to perform basic daily activities, greater dysfunction, and ultimately a higher risk of future falls [11,12]. Although fear of falling is prevalent in older adults in general, especially after a fall, it is particularly relevant in those who have experienced fall-related injury or fracture [9,10].

Together, falls and fear of falling may result in loss of independence, increased need for care, and a markedly negative impact on quality of life [9]. Improving quality of life remains a central therapeutic objective, in line with the World Health Organization’s emphasis on adding life to years, not only years to life [13].

Given these premises, there is a need for simple, standardized instruments for the screening and monitoring of fall risk in patients with T2DM.

The Falls Efficacy Scale—International (FES-I) is a commonly used instrument for quantifying concern about falling. However, before its use in Romania, local psychometric validation is necessary because linguistic, cultural, and clinical–contextual factors may influence how questionnaire items are understood and answered.

Although the FES-I has been validated in several languages and clinical settings, psychometric performance cannot be assumed to be identical across cultures, healthcare environments, and disease-specific populations. Validation in Romanian older adults with T2DM is particularly relevant because fear of falling in this group is influenced not only by aging, but also by diabetes-related complications such as peripheral neuropathy, autonomic dysfunction, impaired mobility, and reduced balance confidence.

The aim of this study was to validate the Romanian version of the FES-I in older adults with T2DM and to examine its reliability, construct validity, agreement, and clinical discriminative performance in this target population.

## 2. Material and Methods

### 2.1. Study Design

**Figure 1:**
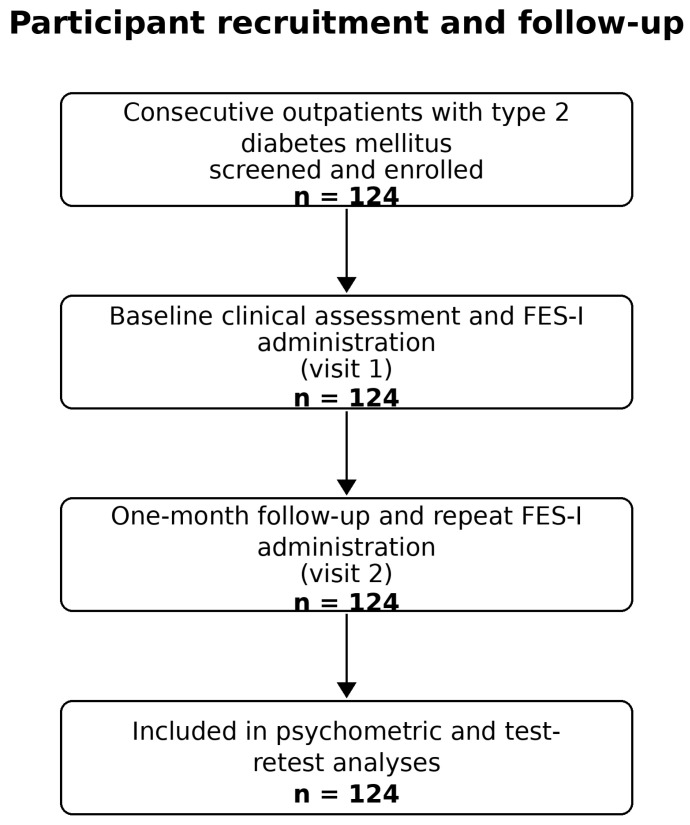
Flow diagram of participant recruitment and follow-up.

The validation study was a prospective, observational, non-interventional, monocenter study conducted in older adults with T2DM. A total of 124 consecutive outpatients aged over 60 years were enrolled using a consecutive-case recruitment approach. Participants were evaluated at an initial study visit and were reassessed at a short follow-up visit scheduled one month later for test–retest reliability analyses of the FES-I instrument. To be eligible, participants had to have a history of T2DM of at least six months before inclusion and to be evaluated in an outpatient setting. Exclusion criteria included unwillingness to provide written informed consent, inability to perform the functional tests or provide reliable medical history, the presence of non-diabetic neuropathies, recent major cardiovascular events, or other conditions considered by the investigators to potentially bias balance-related testing. Participant recruitment and follow-up are summarized in Figure 1.

The study was conducted in accordance with the Declaration of Helsinki, with the study protocol and informed consent form being approved by the Ethics Committee of the “Victor Babeș” University of Medicine and Pharmacy, Timișoara, Romania (Approval Number 27/5 July 2023). Written informed consent was obtained from all participants prior to enrollment.

### 2.2. Translation and Cultural Adaptation

Because this study was designed as a psychometric validation of the Romanian language FES-I rather than as a de novo translation study, we did not undertake a separate forward–backward translation process within the present project. Instead, the Romanian wording available to the investigators was reviewed before study initiation for clarity, semantic consistency, and applicability to older Romanian adults with T2DM. The questionnaire was then administered in the same standardized Romanian form throughout the study, without item modification during data collection.

### 2.3. Patients

A total of 124 participants were included in the validation study. The cohort had a mean age of 71.0 ± 5.9 years, and 57.3% of participants (71 individuals) were women. Mean BMI was 31.8 ± 4.4 kg/m^2^ and mean diabetes duration was 10.5 ± 6.7 years. The mean Berg Balance Scale (BBS) score was 42.1 ± 9.4, mean Timed Up and Go (TUG) time was 12.9 ± 4.4 s, and mean single-leg stance (SLS) time was 8.6 ± 10.2 s. Baseline characteristics are presented in Table 1.

### 2.4. Clinical, Anthropometric and Laboratory Assessments

A complete and structured medical interview was performed by the same evaluator, including diabetes history, comorbidities, and current therapies, using a standardized data collection form. Anthropometric parameters (including weight and height for body mass index calculation) were measured in standardized conditions. Laboratory tests (including HbA1c and routine metabolic and inflammatory parameters) were performed in the same laboratory using standardized methods and calibration procedures. Peripheral diabetic neuropathy status was defined using the Michigan Neuropathy Screening Instrument (MNSI), which includes a symptom questionnaire (MNSI-A) and an objective clinical examination component (MNSI-B). To reduce between-observer variability, evaluations were performed by the same operator in the same examination setting

### 2.5. Balance, Mobility and Fear-of-Falling Assessment

Fear of falling was quantified using the Falls Efficacy Scale—International (FES-I), a 16-item self-report questionnaire assessing concern about falling during basic and more demanding daily activities. Each item is rated on a four-point Likert scale (1 = not at all concerned; 4 = very concerned), with higher total scores indicating greater fear of falling and reduced confidence in balance. The FES-I was administered twice—at baseline (FES-I v1) and at the one-month follow-up (FES-I v2)—to evaluate test–retest reliability and agreement. For construct validity, additional measures were collected: Fear-of-Falling Questionnaire—Revised (FFQ-R), a multidimensional fear-of-falling measure; Berg Balance Scale (BBS), a performance-based balance scale (0–56), with lower scores indicating worse balance; Timed Up and Go (TUG), recorded in seconds, with higher values indicating worse mobility; Single-Leg Stance (SLS), recorded as the time (seconds) the participant maintained single-leg stance, with higher values indicating better static balance.

### 2.6. Statistical Analysis

Data were collected and analyzed using the MedCalc Statistical Software v.23.5.21 (MedCalc Software Ltd., Ostend, Belgium). Psychometric and receiver operating characteristic analyses were additionally implemented in R v.4.5.3 (R Foundation for Statistical Computing, Vienna, Austria), using the following packages: psych (internal consistency, omega, descriptive item statistics), irr or psych (ICC), BlandAltmanLeh (Bland–Altman plots and limits of agreement), pROC (ROC curves and AUC with confidence intervals), GPArotation (factor rotation), and psych/paran (parallel analysis). Figures were generated using ggplot2 v.3.4.3. The statistical significance threshold was set at α = 0.05 (two-tailed). For families of related tests, false-discovery-rate adjustment (Benjamini–Hochberg) was used as a sensitivity analysis. Prior to analysis, all variables were tested for normality using the Shapiro–Wilk method. Continuous variables with Gaussian distribution were reported as mean ± standard deviation, while non-normally distributed variables were reported as median (interquartile range). Categorical variables were reported as absolute frequencies and percentages. Group comparisons used the unpaired *t*-test or Mann–Whitney U test (two groups) and one-way ANOVA or Kruskal–Wallis test (three or more groups), as appropriate; categorical comparisons used the chi-squared test.

### 2.7. Internal Consistency Analysis

Item performance was assessed using the response distribution across the four Likert categories, missingness per item, floor and ceiling effects for items and total score (considered relevant if more than 15% of participants achieved the minimum or maximum score), as well as corrected item–total correlations. Internal consistency reliability was evaluated using Cronbach’s alpha (α), including alpha if item deleted. As a robustness analysis, McDonald’s omega total (ω) was also estimated. Internal consistency was computed separately for FES-I v1 and v2.

### 2.8. Test–Retest Reliability and Agreement

Test–retest reliability between FES-I v1 and v2 was evaluated using the intraclass correlation coefficient (ICC), with a two-way random-effects model and absolute agreement, appropriate for repeated measurements where both the participants and measurement occasions are considered random. ICC values were interpreted using commonly accepted thresholds (poor < 0.50, moderate 0.50–0.75, good 0.75–0.90, excellent > 0.90). In addition, Spearman’s correlation was reported for monotonic association.

Agreement was evaluated using Bland–Altman analysis (mean difference/bias and 95% limits of agreement). Heteroscedasticity was assessed visually and by correlating absolute differences with the mean of paired measurements; if present, analyses using log-transformed scores or regression-based limits were planned as sensitivity analyses.

### 2.9. Construct Validity

Construct validity was evaluated using pre-specified hypotheses regarding the direction and magnitude of correlations: higher FES-I was expected to correlate positively with FFQ-R, TUG, PHQ-9 and GAD-7, and negatively with BBS, SLS, and MoCA. Correlations were primarily assessed using Spearman’s rho due to the ordinal structure of FES-I items and the expected non-normality of some variables. Correlation strength was interpreted as weak (<0.30), moderate (0.30–0.59), and strong (≥0.60).

### 2.10. Discrimination Analyses

Receiver operating characteristic (ROC) analyses evaluated the ability of FES-I to discriminate participants with clinically relevant impairment, using objective anchors such as BBS below a fall-risk-known threshold and TUG above a mobility–impairment threshold. Area under the ROC curve (AUC) with 95% confidence intervals was reported. The optimal FES-I cut-off was identified using Youden’s J statistic, and corresponding sensitivity, specificity, and likelihood ratios were computed.

## 3. Results

### 3.1. Score Distribution and Descriptive Properties

**Figure 2:**
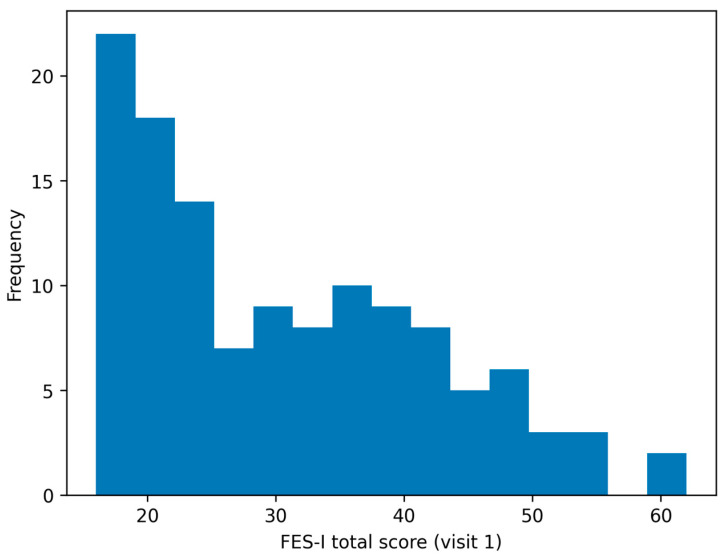
Distribution of the FES-I total score at the baseline (v1) visit. Higher scores indicate greater concern about falling.

The mean FES-I total score at Visit 1 (v1) was 30.8 ± 11.4 (median 29.0, IQR 21.0–40.0; range 16–62). At the one-month follow-up (v2), the mean total score was 31.1 ± 11.6 (median 30.5, IQR 20.8–39.0; range 16–62). The mean change (v2-v1) was 0.31 ± 2.70 points and did not differ significantly from zero (paired *t*-test *p* = 0.209). Floor effects were modest (score = 16: 8.9% at v1 and 10.5% at v2) and no ceiling effects were observed. The observed floor effects remained below the conventional 15% threshold that was considered clinically relevant, supporting the acceptable score distribution at the total-score level. The score distribution and descriptive properties of the FES-I score are presented in Table 2 and Figure 2.

### 3.2. Internal Consistency

The Romanian FES-I instrument demonstrated excellent internal consistency, with a Cronbach’s alpha of 0.945 at Visit 1 and 0.947 at follow-up. Corrected item–total correlations at baseline ranged from 0.540 to 0.799. Alpha if item deleted remained high for all items (range 0.939–0.944).

### 3.3. Test–Retest Reliability and Agreement

**Figure 3:**
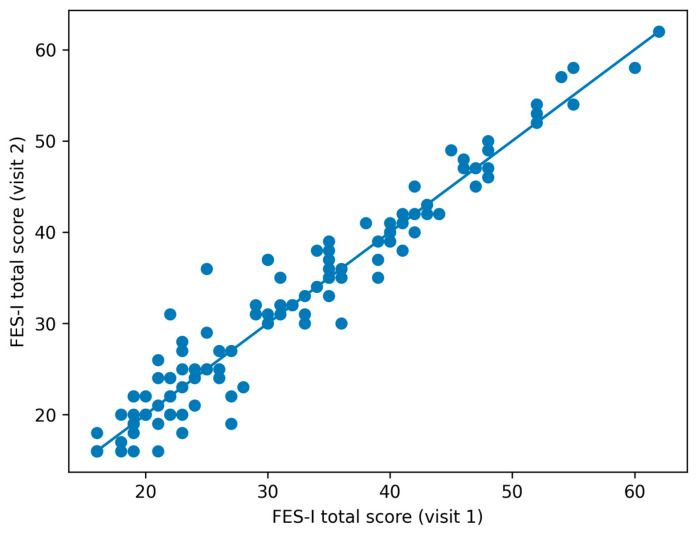
Correlation between FES-I scores recorded at Visit 1 and Visit 2. The graph illustrates the high test–retest consistency of the total score.

**Figure 4:**
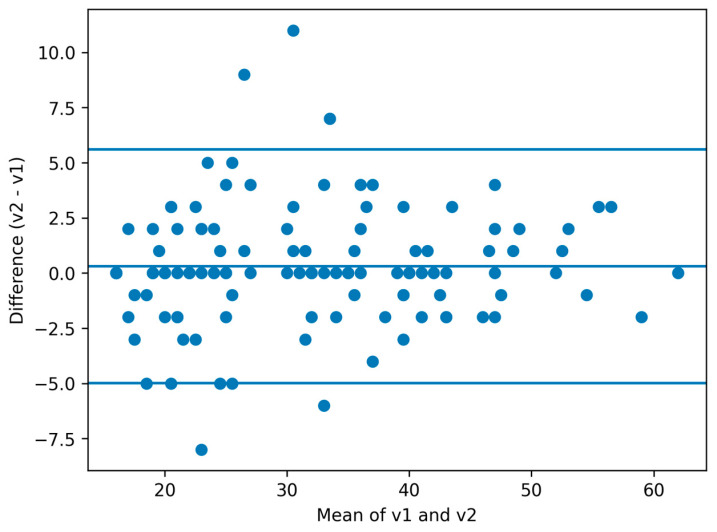
Bland–Altman plot for agreement analysis of FES-I scores at Visit 1 and Visit 2. The central line represents the mean difference, and the outer lines indicate the 95% limits of agreement.

Test–retest reliability of the total score across the one-month interval was excellent, with an ICC of 0.972 (95% CI 0.956–0.983). Correlations between v1 and v2 were very high (Pearson r = 0.973, Spearman rho = 0.961; both *p* < 0.001; Figure 3), and Lin’s concordance correlation coefficient was 0.972. Bland–Altman analysis (Figure 4) showed a small mean bias of 0.31 points, with 95% limits of agreement from −4.98 to 5.60 points.

The standard error of measurement (SEM) for the total score was 1.89 points. The minimal detectable change at the 95% confidence level (MDC95) was 5.25 points, indicating that an individual change of approximately six points or more is unlikely to be due to measurement error alone.

### 3.4. Construct Validity

The validity of the construct for the FES-I instrument was supported by strong associations with related patient-reported and performance-based measures. At Visit 1, FES-I correlated strongly with FFQ-R (Spearman rho = 0.787) and moderate-to-strong objective functional performance (BBS rho = −0.631; TUG rho = 0.547; SLS rho = −0.498; all *p* < 0.001). FES-I also showed moderate correlations with depressive and anxiety symptoms (PHQ-9 rho = 0.638; GAD-7 rho = 0.585; both *p* < 0.001) and an inverse association with cognitive performance (MoCA rho = −0.300, *p* < 0.001).

### 3.5. Diagnostic Accuracy for Identifying Functional Impairment

**Figure 5:**
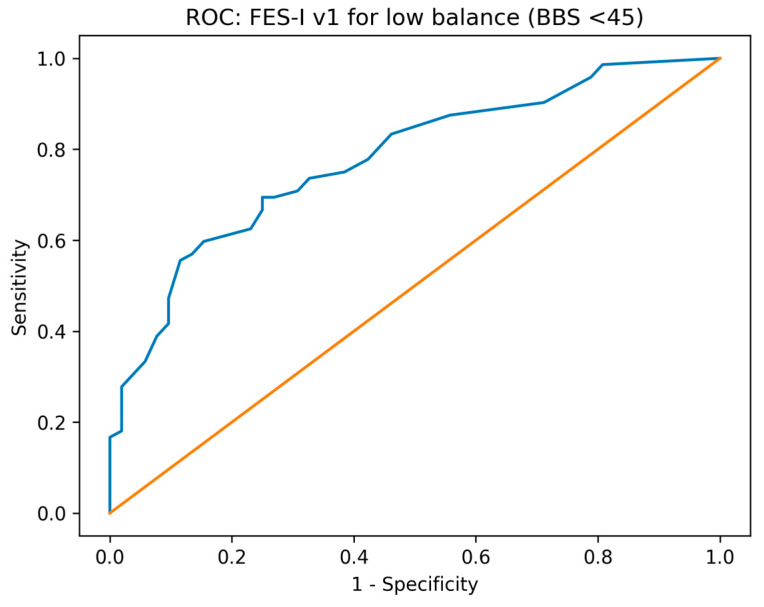
ROC curve of FES-I for identifying low balance (BBS < 45).

**Figure 6:**
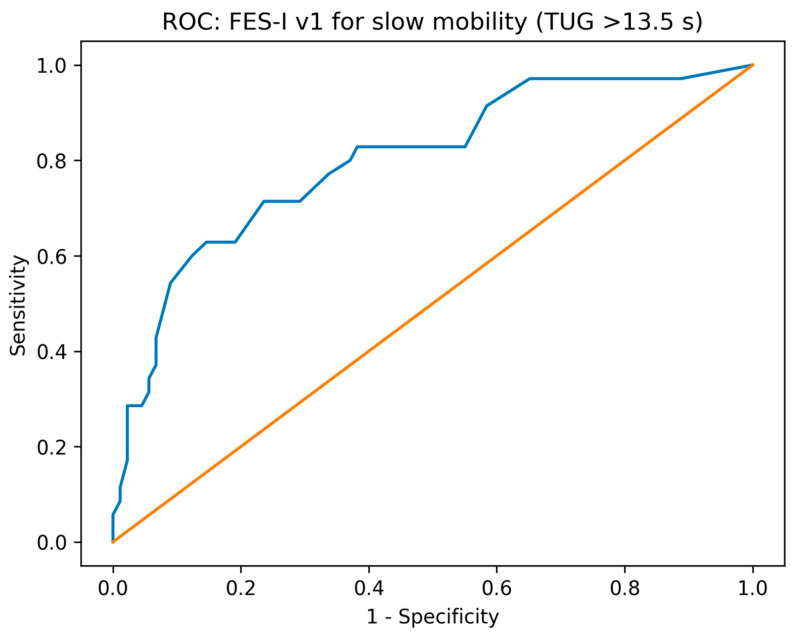
ROC curve of FES-I for identifying slow mobility (TUG > 13.5 s).

In ROC analyses (Figure 5 and Figure 6), baseline FES-I demonstrated good discrimination for identifying low balance performance (BBS < 45; AUC = 0.779, 95% CI 0.699–0.859). The Youden-optimal cut-off was 29 points (sensitivity 0.694, 95% CI 0.580–0.789; specificity 0.750, 95% CI 0.618–0.848). FES-I also discriminated slow mobility defined as TUG > 13.5 s (AUC = 0.800, 95% CI 0.705–0.895), with an optimal cut-off of 39 points (sensitivity 0.629, 95% CI 0.463–0.768; specificity 0.854, 95% CI 0.766–0.913). Detailed ROC analyses are presented in Table 3.

### 3.6. Dimensionality

Sampling adequacy for factor extraction was excellent (KMO = 0.928; individual KMO range 0.901–0.956). Bartlett’s test of sphericity was significant (chi-square = 1394.4, df = 120.0, *p* < 0.001), supporting factorability. Principal component analysis indicated a dominant first component (eigenvalue 8.613), accounting for 57.8% of total variance; only the second component exceeded the Kaiser criterion (eigenvalue 1.272).

## 4. Discussions

### 4.1. Relevance of the Findings

The results of this study support the use of the Romanian FES-I as a clinically useful instrument in both routine care and research among older adults with T2DM. The scale showed excellent internal consistency, excellent short-term test–retest reliability, and good agreement, indicating that it provides stable measurement of concern about falling in this target population.

Construct validity was also supported by the observed correlations with FFQ-R and with performance-based measures such as BBS, TUG, and SLS. These findings suggest that higher FES-I scores reflect not only subjective concern about falling but also clinically relevant impairment in balance and mobility. In this context, the Romanian FES-I may be particularly helpful for identifying patients who would benefit from more comprehensive fall-risk assessment and targeted preventive interventions.

### 4.2. Other Studies

Previous studies have shown that FES-I has good psychometric performance across different linguistic and clinical settings, including community-dwelling older adults, rehabilitation cohorts, and neurological populations with balance impairment [14]. However, the populations, clinical contexts, and validation aims of these studies differ from those of the present research.

Unlike prior validations conducted in broader geriatric or neurological samples, the present study specifically examined Romanian older adults with T2DM receiving outpatient care. This distinction is clinically important because the fear of falling in diabetes may be shaped by disease-specific mechanisms, including peripheral neuropathy, autonomic dysfunction, reduced proprioception, slower gait, and impaired balance confidence. Therefore, the present study adds language-specific and disease-specific evidence rather than duplicating previous generic validations.

Our findings are broadly consistent with earlier reports, showing strong internal consistency and good validity of the FES-I, as well as clinically meaningful associations between fear of falling and objective mobility measures. The correlations observed with TUG and BBS support the relevance of the scale for identifying functional vulnerability, while the association with FFQ-R further supports convergent validity [15,16,17].

Taken together, these comparisons indicate that the Romanian FES-I performs in a manner consistent with the international literature while also addressing an unmet need for validation in a specific high-risk population of older adults with diabetes.

### 4.3. Strengths and Limitations of the Study

The main strength of this study is that it provides a Romanian language psychometric validation of the FES-I in a clinically vulnerable population in whom fear of falling is highly relevant. The instrument may facilitate rapid screening, support more comprehensive balance assessment, and help identify patients who may benefit from both fall-prevention strategies and physical or psychological rehabilitation.

Several limitations should be acknowledged. First, this was a monocentric study with a moderate sample size, which may limit representativeness. As participants were consecutive outpatients recruited from a single center, the cohort may not fully represent all Romanian older adults with T2DM, particularly those from rural settings, institutionalized populations, or patients with more severe disability. Second, only older adults with T2DM were included; therefore, the findings should not be generalized to younger individuals, non-diabetic elderly adults, or other linguistic and cultural settings without further validation. Third, the study did not include a non-diabetic elderly control group, so it does not allow direct conclusions regarding whether baseline fear of falling is specifically attributable to diabetes or diabetes-related complications. Fourth, the follow-up interval was limited to one month. Although this duration is appropriate for assessing short-term test–retest reliability, it does not allow evaluation of long-term stability, responsiveness to intervention, or predictive validity for future falls. Finally, although approximately one in ten participants achieved the minimum FES-I score, the observed floor effect remained below the conventional 15% threshold considered clinically relevant; however, in low-risk individuals with minimal concern about falling, the instrument may be less sensitive to detecting further improvement.

Future multicenter studies with longer follow-up and incident-fall assessment should evaluate predictive validity, responsiveness to intervention, and performance in broader Romanian populations.

## 5. Conclusions

The FES-I instrument demonstrated excellent psychometric performance in older Romanian adults with T2DM. The instrument showed high internal consistency and excellent short-term test–retest reliability over a one-month period, indicating stable measurement of concern about falling in this clinical population. Measurement error was low, and the derived minimal detectable change provides a practical threshold for interpreting whether within-person score differences are likely to reflect true clinical change rather than random variation.

The Romanian FES-I is a reliable and valid measure for quantifying fear of falling in people with diabetes and can be used in both clinical practice and research, including longitudinal monitoring and evaluation of interventions. Its interpretation should, nevertheless, remain anchored to the target population in which it was validated, namely older Romanian adults with T2DM.

## Figures and Tables

**Table 1:** Baseline characteristics of the study cohort. Values are presented as mean ± SD or *n* (%), as appropriate. Abbreviations: BMI, body mass index; BBS, Berg Balance Scale; TUG, Timed Up and Go; SLS, single-leg stance; FFQ-R, Fear-of-Falling Questionnaire—Revised.

Characteristic	Value
Participants, *n*	124
Age, years	71.0 ± 5.9
Female sex, *n* (%)	71 (57.3%)
Urban residence, *n* (%)	69 (55.6%)
BMI, kg/m^2^	31.8 ± 4.4
Waist circumference, cm	111.8 ± 9.7
Diabetes duration, years	10.5 ± 6.7
Orthostatic hypotension, *n* (%)	51 (41.1%)
BBS score	42.1 ± 9.4
TUG, seconds	12.9 ± 4.4
Single-leg stance (SLS), seconds	8.6 ± 10.2
FFQ-R score	20.1 ± 15.6

**Table 2:** Descriptive statistics for FES-I scores at test and retest measurements. FES-I scores are reported as mean ± SD, median (IQR), and range. Floor and ceiling effects refer to the proportion of participants with the minimum and maximum possible total score, respectively.

Metric	Mean ± SD	Median (IQR)	Range
FES-I total score (v1)	30.8 ± 11.4	29.0 (21.0–40.0)	16–62
FES-I total score (v2)	31.1 ± 11.6	30.5 (20.8–39.0)	16–62
Change (v2-v1)	0.31 ± 2.70	0.0 (−1.0–2.0)	−8–11
Floor effect (score = 16)	v1: 8.9%; v2: 10.5%		
Ceiling effect (score = 64)	v1: 0.0%; v2: 0.0%		

**Table 3:** ROC analysis of FES-I v1 for identifying functional impairment. AUC values are shown with 95% confidence intervals. Sensitivity and specificity are reported for the Youden-optimal cut-off, together with their 95% confidence intervals. Low balance was defined as BBS < 45, and slow mobility as TUG > 13.5 s.

Reference Outcome	AUC (95% CI)	Optimal FES-I Cut-Off	Sensitivity (95% CI)	Specificity (95% CI)	Cases (*n*)	Controls (*n*)
Low balance (BBS < 45)	0.779 (0.699–0.859)	29	0.694 (0.580–0.789)	0.750 (0.618–0.848)	72	52
Slow mobility (TUG > 13.5 s)	0.800 (0.705–0.895)	39	0.629 (0.463–0.768)	0.854 (0.766–0.913)	35	89

## Data Availability

The data presented in this study are available on request from the corresponding author. The data are not publicly available due to local privacy and data protection regulations.

## Abbreviations

The following abbreviations are used in this manuscript:

DN	Diabetic neuropathy
T2DM	Type 2 Diabetes Mellitus
FES-I	Falls Efficacy Scale—International
BBS	Berg Balance Scale
TUG	Timed Up and Go
SLS	Single-leg stance
MNSI	Michigan Neuropathy Screening Instrument
FFQ-R	Fear-of-Falling Questionnaire—Revised
ICC	Intraclass correlation coefficient
ROC	Receiver operating characteristic
AUC	Area under the ROC curve
SEM	Standard error of measurement
MDC95	Minimal detectable change at the 95% confidence level
